# The Relationship between Gender, Severity of Disease, Treatment Type, and Employment Outcome in Patients with Inflammatory Bowel Disease in Israel

**DOI:** 10.1155/2018/5090849

**Published:** 2018-09-09

**Authors:** Timna Naftali, Adi Eindor-Abarbanel, Nahum Ruhimovich, Ariella Bar-Gil Shitrit, Fabiana Sklerovsky-Benjaminov, Fred Konikoff, Shay Matalon, Haim Shirin, Yael Milgrom, Tomer Ziv-Baran, Efrat Broide

**Affiliations:** ^1^Department of Gastroenterology and Hepatology, Meir Medical Center, affiliated with the Sackler School of Medicine, Tel Aviv University, Tel Aviv, Israel; ^2^The Kamila Gonczarowski Institute for Gastroenterology and Liver Diseases, Assaf Harofeh Medical Center, affiliated with the Sackler School of Medicine, Tel Aviv University, Tel Aviv, Israel; ^3^Digestive Diseases Institute, Shaare Zedek Medical Center, Jerusalem, Israel; ^4^Department of Epidemiology and Preventive Medicine, School of Public Health, Sackler Faculty of Medicine, Tel Aviv University, Tel Aviv, Israel

## Abstract

**Introduction:**

Since individuals with IBD typically experience symptoms during their prime years of employment, it raises the question about IBD impact on employment status. Most studies concentrated on absenteeism from work with varying results in different populations. However, absenteeism reflects only one dimension of the ability to work and does not expose the problem of inability to hold a full-time job.

**Aims:**

To evaluate the influence of IBD on unemployment and working hours in Israel. Secondary aims were to investigate the correlation between working hours and the type of medical treatment and the impact of severity of disease.

**Patients and Methods:**

Demographic data, employment status, number of weekly working hours, and disease parameters. The data was compared to that of the general Israeli population extracted from the website of the Central Bureau of Statistics.

**Results:**

242 IBD patients were interviewed. Patients median age was 37.04(IQR 30.23-44.68) years and 88 (36.4%) were men and 154 (63.6%) women. Diagnosis of CD was established in 167 (69%) patients and UC in 65 (26.9%). There was no significant reduction in employment rates or working hours among the IBD patients comparing to the general population. Immunosuppressive or biologic treatment did not influence employment status. The unemployed patients had higher disease severity (median 7.33, IQR 5-10.66) compared to employed patients (median 6, IQR 3.66-7.66; p=0.003).

**Conclusions:**

Although IBD patients in Israel do not have higher unemployment, those with severe disease have lower proportion of employment.

## 1. Introduction

Inflammatory bowel diseases (IBD) are characterized by episodic and continuous symptoms including diarrhea, abdominal discomfort, and fatigue among many other complaints. These symptoms can interfere with the ability to perform daily routine activities [1]. Although few people with IBD experience permanent disability [2], the relapsing-remitting nature of the disease may lead to recurrent hospitalization and periods of disability [3–5]. Additionally, some patients need routine intravenous treatments such as biologics [6, 7] and iron supplementation [8, 9]. These are time-consuming and can challenge IBD patients' ability to meet their obligations to their place of employment.

The onset of IBD can occur at any age [10], but Crohn's disease (CD) is most often diagnosed in patients in their third decade of life and ulcerative colitis (UC) in patients in their fourth decade [11], corresponding with productive work years. Since individuals with IBD typically experience symptoms during their prime years of employment, we evaluated the impact of IBD on employment status. Previous studies have concentrated on absenteeism from work with varying results among different populations [3, 12]. However, absenteeism reflects only one dimension of the ability to work and does not expose the problem of inability to hold a full-time job. Other studies explored employment status of patients, but did not refer to the number of hours worked [3]. Furthermore, only one study investigated the long-term impact of the disease on the ability of IBD patients to work [13].

Israel has the third highest prevalence of IBD in the world and this has nearly doubled in the past decade [14]. At the end of 2015, 38,291 IBD patients were residing in Israel, with a prevalence of 459 per 100,000 (0.46%). Despite this high prevalence, data on its effect on employment status in the Israeli population is lacking. In this study, we evaluated the influence of IBD on unemployment and working hours in Israel. Secondary aims were to investigate the correlation between working hours and the type of medical treatment and disease severity.

## 2. Methods

### 2.1. Participants

Between November 2015 and May 2017, consecutive ambulatory patients, ages 25 to 65 years, with an established diagnosis of IBD were enrolled in the study. Diagnosis of Crohn's disease (CD) and ulcerative colitis (UC) were previously confirmed by established criteria based on clinical, endoscopic, histopathological, and radiological findings. Patients were recruited from three university-affiliated hospitals in Israel: Assaf Harofeh, Meir Medical Center, and Shaare Zedek Medical Center.

All patients completed questionnaires regarding demographic data, employment status, number of weekly working hours, and disease parameters. The study was approved by the Ethics Committee of each hospital. All patients provided signed informed consent.

Data about employment rates and working hours of the general population was extracted from the website of the Central Bureau of Statistics (CBS) of Israel, which was updated in 2016.

Clinical disease severity was calculated based on a scale modified from a study by the GETAID [15] group. This scale was developed by a panel of 20 IBD experts from the “Groupe d'Etudes Thérapeutiques des Affections Inflamma- toires du tube Digestif” (GETAID) who selected the most relevant criteria for discriminating a severe from a mild-to-moderate CD course during a 15-year period. The severity scale was calculated based on an average of disease severity as reported by the physician. The scale was graded on a scale from 0 to 5 (0, no symptoms; 1, mild symptoms; 2, medium symptoms; 3, active disease; 4, hospitalization in the past year; 5, surgery in the past year or current stoma). Another component of severity was based on medical treatment, based on the total score of a scale varying from 0 to 5 (0, no treatment; 1, 5-ASA or antibiotics; 2, less than 10 mg per day dose steroids; 3, steroids in a regular dose; 4, immunomodulatory treatment; 5, biological treatment). These scales were calculated for each year of the last three years. The final score was the average of the score from each year over the last three years (the Appendix).

### 2.2. Data Analysis

Categorical variables were described using frequency and percentage. Continuous variables were evaluated for normal distribution using histograms and Q-Q plots. One sample T- test was used to compare working hours to those reported by the CBS. Working status was compared to the CBS using one sample binomial test. Within the cohort, categorical variables were compared using chi-squared test and Fisher's exact test. Continuous variables were compared using independent simple* t*-test. Spearman's rank correlation coefficient was used to describe the association between continuous variables.

All statistical analyses were performed using IBM SPSS Statistics for Windows, Version 23.0 (Released 2015,IBM Corp. Armonk, NY).

## 3. Results

### 3.1. Patient Population

From November 2015 through May 2017, 242 consecutive IBD patients attending outpatient clinics in the participating hospitals were interviewed. Patients' median age was 37.04(IQR 30.23-44.68) years and 88 (36.4%) were men and 154 (63.6%) women. A total of 167 (69%) patients were diagnosed with CD, 65(26.9%) with UC and 10 (4.1%) with IBD-U and 50 (20%) with perianal disease. Demographic and disease characteristics are listed in Table 1. Four patients did not report employment status. Because of the low rates of refusal, these patients were not analyzed separately.

### 3.2. Employment and Working Hours

Employment status and working hours, according to gender are compared in Table 2.  Table 3 compares working hours between the study patients and the Israeli population according to gender. There were 79 patients on immunosuppressive drugs, and 19% were unemployed, while the rate of unemployment in the other patients was 16.5% (p=0.625). There was no significant difference in the weekly hours worked between these two groups (40.08 ±12.97) and (39.56 ± 13.51, respectively; p=0.809).

Among 116 patients receiving biologic treatment, the unemployment rate was 20.7%, as compared to the other patients whose unemployment rate was 13.9% (p=0.168). There was no significant difference in the working hours between these two groups (39.99 ± 12.58 vs. 39.49 ± 13.89, respectively; p=0.801).

There was a significant difference in disease severity scores between the unemployed patients (median 7.33, IQR 5-10.66) as compared to the employed patients (median 6, IQR 3.66-7.66; p=0.003). There was also a medium correlation between working hours and disease severity (Spearman correlation = 0.368).

## 4. Discussion

Crohn's disease and UC affect nearly 0.5% of the Israeli population [5]. The relapsing-remitting disease course and the complications are major sources of morbidity [3, 16]. The diseases are associated with work absenteeism and constitute substantial economic burden to employers [17].

Surprisingly, we found that both employment status and working hours of IBD patients did not differ from those of the general population. These results are similar to the findings of the Danish cohort published by Vester-Andersen et al. [3], but were in contrast with the study of Mahlich et al. [4] from Japan who described a lower employment rate in IBD patients as compared to the general population. When divided according to age groups, Malich [4] observed that older IBD patients have lower employment rates than does the general population. Vester-Andersen also demonstrated that patients with IBD were at higher risk for work disability, particularly male patients older than 55 years-of-age. We also found that older male patients worked fewer hours per week, although this trend did not reach statistical significance because of the small sample size.

Although employment among IBD patients overall equaled that of the general population, this was not true for the subgroup of patients with severe disease. Disease severity was significantly correlated with rates of unemployment and with fewer hours worked per week. Perianal Crohn's disease as a separate severity measure was recently reported to be associated with unemployment in Crohn's disease. When correcting for age, disease duration, inflammatory bowel disease-related surgery, and faecal incontinence, active perianal disease was independently affecting employment (OR 0.67; 95% CI 0.50–0.91;* P *= 0.01). Although in our study we did not consider any perianal disease, we did include complicated perianal disease requiring surgery as a component in the disease severity scale [18].

This study used a comprehensive method to evaluate disease severity by using a modified calculator from the GETAID [6] group. It considers the 3-year course of the disease instead of a certain time frame. We preferred this approach because of the assumption that employment status is influenced by a long period of time prior to that currently being investigated. Therefore, a score that reflects disease severity over a prolonged period is superior to a score that reflects disease activity over one week [19].

Although we demonstrated that more severe disease is correlated with unemployment, immunosuppressive or biologic treatment was not correlated with unemployment among IBD patients. Biologic treatment sometimes entails IV administration and may therefore cause absenteeism. However, it may induce remission and prevent permanent compromise of work ability.

The current study has some limitations. Only ambulatory patients were included; thus, there is a possibility of selection bias, because adherent patients who attend out-patients clinics may have better rates of employment than nonadherent patients do. Also, we included only patients 25 years and older because we wanted to compare the results with the data available from the CBS. Finally, we did not differentiate between patients receiving medications intravenously [20, 21] or subcutaneously [22] because the groups were too small to analyze separately. As this study investigated permanent effects on unemployment rather than absenteeism, we assumed all biologic treatments can have an effect due to adverse reactions [23–25] and the inconvenience of the route of administration.

This study is the first to investigate the roll of IBD on work habits among Israeli IBD patients. We demonstrated that although these patients do not have higher rates of unemployment, those with severe disease have a lower proportion of employment. In addition, the type of drug administered did not affect employment rates. Therefore, health providers in Israel should pay more attention to evaluating disease severity and increase their treatment efforts when facing a patient with sever disease. The aim of treatment should be not only to improve patients' symptoms, but also to lower unemployment rates.

## Figures and Tables

**Table 1:** Demographic and disease characteristics.

**Characteristic**	**Patients N=242 **
Age median(IQR)	37.04(30.23-44.68)
**Gender n(%)**	
Male	88(36.4)
Female	154 (63.2)
Education >12 yrs	154(63.3)
**Marital status n(%)**	
Single	52(21.5)
In a relationship	171(70.7)
Past relationship	17(7)
**Income level n(%)**	
Low	126(54.3)
Medium	46(19.8)
High	60(25.9)
**Disease type**	
Crohn's	167(69)
UC	65(26.9)
IBDU	10(4.1)
**Other diseases n(%)**	
Diabetes	10(4.1)
Cardiovascular	4(1.7)
Hypertension	10(4.1)
Cancer	2(0.8)
Lung	3(1.2)
Hyperlipidemia	13(5.4)
Other disease	26(10.7)
Disease severity score	6(3.66-8)
**Medications n(%)**	
Topical 5_ASA	24(9.9)
Tab 5_ASA	80(33.1)
Budesonide	12(5)
Prednisone	19(7.9)
Immunosuppressive	80(33.2)
Biologics	118(48.8)
Cannabis	22(9.1)
More than 3 medications	53 (21.9)

**Table 2:** Comparison of the proportion of employment among IBD patients and the general Israeli population.

**Gender**	**Israeli citizen**	**Study patients**	**p-value**	**Crohn's**	**p-value**	**UC**	**p-value**
Men	81.4	89.5	<0.001	92.1	<0.001	78.9	0.482

Women	72	78.9	0.043	76.2	0.201	86.7	0.021

**Table 3:** Comparison of hours worked per week between patients and the general population.

Age group	Men				Women			
	Patients, mean (SD)	N	General population, mean	P-value	Patients mean (SD)	N	General population, mean	P-value
25-34.9	43.85 (10.74)	32	43.9	0.9	37.44 (11.7)	55	36.4	0.51

35-44.9	46.75 (12.31)	24	46.9	0.953	37.67 (15.42)	34	37.5	0.947

45-54.9	40 (19.27)	10	46.5	0.314	33.33 (13.58)	15	36.9	0.327

55-64.9	38.33 (9.31)	6	44.1	0.19	39 (9.76)	7	34.5	0.268

**Table 4:** Disease severity questionnaire.

	**Year:**		
**Disease activity:**	**This year**	**One year ago**	**Two years ago**
No Symptoms-0			

Mild Symptoms-1			

Medium Symptoms-2			

Active Disease- 3			

Hospitalization- 4			

Surgery- 5			

Stoma- 5			

**(a) Highest score**			

**Treatment:**			

No Treatment-0			

5-ASA or Antibiotics-1			

Low dose steroids-2			

Steroids-3			

Immunosuppresive-4			

Biologics-5			

**(b) Sum of treatments**			

**Total year score (a+b)**			

## Data Availability

The data used to support the findings of this study are available from the corresponding author upon request.

## Appendix

See Table 4.
